# Fine-tuning management of the Heart Assist 5 left ventricular assist device with two- and three-dimensional echocardiography

**DOI:** 10.5830/CVJA-2015-083

**Published:** 2016

**Authors:** Zumrut Tuba Demirozu, Deniz Suha Kucukaksu, Nurcan Arat

**Affiliations:** Department of Heart Transplant and Mechanical Circulatory Support, Istanbul Bilim Medical Faculty, Sisli Florence Nightingale Hospital, Istanbul, Turkey; Department of Cardiovascular Surgery, Koc University Hospital, Istanbul, Turkey; Department of Heart Transplant and Mechanical Circulatory Support, Istanbul Bilim Medical Faculty, Sisli Florence Nightingale Hospital, Istanbul, Turkey; Department of Cardiology, Istanbul Bilim Medical Faculty, Sisli Florence Nightingale Hospital, Istanbul, Turkey

**Keywords:** Heart Assist 5 LVAD, continuous-flow pumps, two- and three-dimensional echocardiography

## Abstract

**Introduction:**

Left ventricular assist device (LVAD) implantation is a viable therapy for patients with severe end-stage heart failure, providing effective haemodynamic support and improved quality of life. The Heart Assist 5 (Micromed Cardiovascular Inc, Houston, TX) continuous-flow LVAD has been on the market in Europe since May 2009.

**Methods:**

We evaluated nine Heart Assist 5 LVAD patients with two- and three-dimensional transthoracic echocardiographic (TTE) and transoesophageal echocardiographic (TEE) parameters between December 2011 and December 2013. The pre-operative TTE LVAD evaluations included left ventricular (LV) function and structure, quantification of right ventricular (RV) function and tricuspid regurgitation (TR), assessment of aortic and mitral regurgitation, and presence of patent foramen ovale and intra-cardiac clots. Peri-operative TEE determined the inflow cannula and septum position, and assessed the de-airing process while weaning from cardiopulmonary bypass. Post-operative serial follow-up TTE showed the surgical results of LVAD implantation, determined the overall structure and function of the LV, RV and TR, and observed the inflow and outflow cannula position.

**Results:**

Nine patients who had undergone Heart Assist 5 LVAD implantation and had been followed up for more than 30 days were included in this study. Eight patients had ischaemic cardiomyopathy and one had adriamycin-induced cardiomyopathy. Pre-implantation data: the mean age of the patients was 52 ± 13 (34–64) years, mean body surface area (BSA) was 1.8 ± 0.2 (1.6–2.0) m2, mean cardiac index (CI) was 2.04 ± 0.4 (1.5–2.6) l/min/m2, mean cardiac output (CO) was 3.7 ± 0.7 (2.6–4.2) l/min, mean ejection fraction (EF) was 23 ± 5 (18–28)%, and right ventricular fractional area contraction (RVFAC) was 43 ± 9 (35–55)%. One patient had aortic valve replacement (AVR) during the LVAD implantation, and excess current alarms and increased power were suspected to be caused by a possible thrombus. Close follow up with TTE studies were carried out to clear the LV of thrombus formation, and the inflow cannula position was checked to maintain the septum in the midline, so preventing the suction cascade. Four patients were followed up for more than two years, and two were followed up for more than a year. Three patients died due to multi-organ failure. Follow-up speed-change TTE studies of six patients showed that the mean speed was 9 800 ± 600 (9 500–10 400) rpm, and mean CO was 4.7 ± 0.3 (4.3– 5.0) l/min during the three-month post-implant period.

**Conclusion:**

We believe that TTE can play a major role in managing LVAD patients to achieve optimal settings for each patient. A large series is mandatory for assessment of echocardiographic studies on Heart Assist 5 LVAD.

## Introduction

Left ventricular assist devices (LVADs) are the accepted treatment modality for advanced end-stage heart failure patients.^1^,^2^,^3^,^4^,^5^ LVADs have been reported to be effective therapy in supporting cardiovascular circulation and end-organ function for weeks and even years. 1,2,3 Increased use of LVAD therapy for patients with advanced heart failure has revealed a new paradigm for this novel physiology.

It has been reported that there is a knowledge gap on these new-generation, continuous-flow pumps and their effect on cardiac and end-organ physiology. There is a growing need for practising heart failure cardiologists and transplant surgeons who take care of these patients to evaluate the pumps and their effects in patients.^1^,^2^,^3^,^4^,^5^,^6^,^7^ Monitoring, tracking and management of these patients needs experience so that the pumps work more efficiently with minor side effects and complications.

Echocardiography is the most important non-invasive imaging tool for LVAD assessment at pre-, peri- and post- implant, and during long-term follow up.^1^,^2^,^3^,^4^,^5^,^6^,^7^,^8^ Serial, non-invasive echocardiographic studies would help management strategies of these patients and identify mechanical dysfunction timeously.

**Fig. 1:**
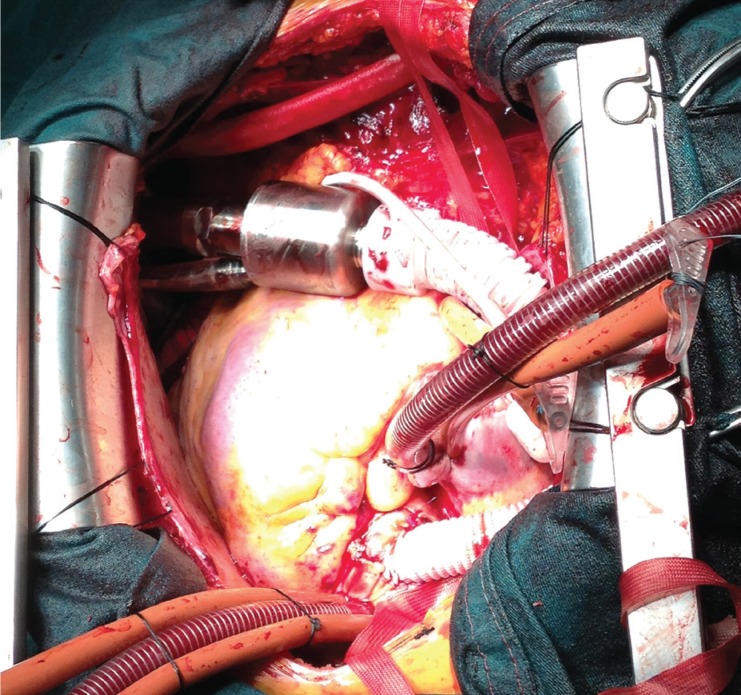
Heart Assist 5 LVAD implantation.

**Fig. 2:**
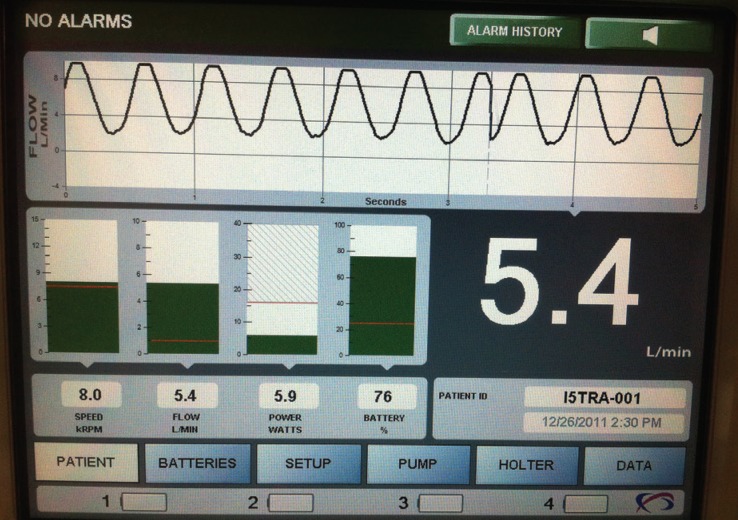
Heart Attendant, the portable console.

**Fig. 3:**
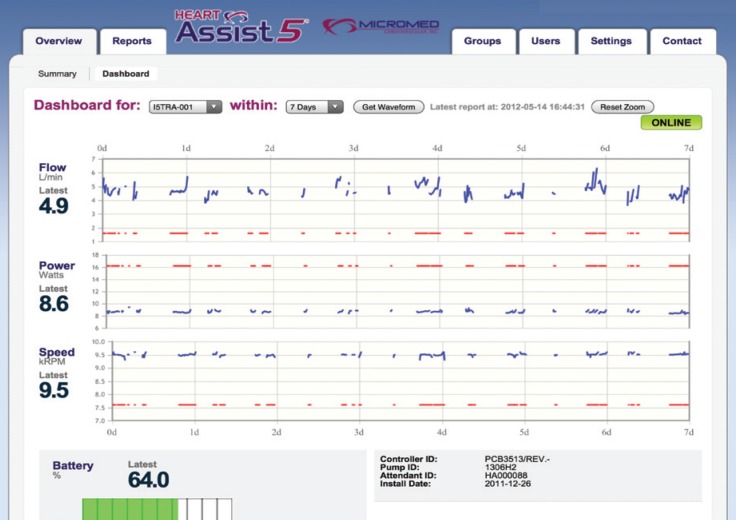
Remote monitoring of Heart Assist 5.

The Heart Assist 5 LVAD (Micromed Cardiovascular Inc, Houston, TX) is small and compact, weighing 92 g and can easily be implanted above the diaphragm (Fig. 1). The Heart Assist 5 flow probe, an implantable ultrasonic flow, measures the blood flow quite accurately while blood is pumped through the outflow graft to the aorta. Changes in pump speed and electronic current provide important information about the volume of blood in the chambers and decompression of the heart, and thus minor and major side effects can easily be detected. Also remote monitoring of these patients helps with patient care, as the primary physicians are informed either by cellphone message or e-mail notification about marginal haemodynamic changes in LVAD parameters (Fig. 2)(Fig. 3)

Although extensive data have been reported in the literature concerning echocardiographic evaluation of other continuousflow pumps, there are no data on the Heart Assist 5 LVAD and its long-term outcomes. The aim of our study was to describe this small, new-generation pump and the first implantation experience from Istanbul, Turkey.

## Methods

From December 2011 to December 2013, Heart Assist 5 LVADs were implanted in nine patients at our hospital. Eight patients had ischaemic cardiomyopathy and one had adriamycin-induced cardiomyopathy. The mean age of the patients was 53 ± 13 (34–64) years Table 1..

**Table 1:** Demographic and pre-implant characteristics

*Parameters*	*Number of patients and mean ± SD*
Gender	
Men	5
Women	4
Age, years	52 ± 13
Weight, kg	74 ± 12
Body surface area, m^2^	1.8 ± 0.2
Heart disease	
Ischaemic cardiomyopathy	8
Adriamycin-induced cardiomyopathy	1
Purpose of LVAD	
Bridge to transplantation	9
Diabetes mellitus	3
Hypertension	3
AICD-CRT-D implantation	9
Previous cardiac surgery	
CABG	1
Pre-implant support	
Intra-aortic balloon pump	1
Haemodynamic values	
Left ventricular ejection fraction, %	23 ± 5
Cardiac index, l/min/m^2^	2.04 ± 0.4
Cardiac output, l/min	3.8 ± 0.7
PCWP, mmHg	30 ± 8
Coagulation profile	
Prothrombin time, sec	17.3 ± 7.5
International normalised ratio	1.58 ± 0.6
Partial thromboplastin time, sec	33.9 ± 2.8
Laboratory data	
BUN, mg/dl	22 ± 10
Creatinine, mg/dl	1.17 ± 0.3
Na, mEq/l	136 ± 2
Albumin, g/l	3.8 ± 0.5
Alkaline phosphatase, U/l	73 ± 25
SGOT, U/l	18 ± 10
SGPT, U/l	26 ± 23
Total bilirubin, mg/dl	0.92 ± 0.5

SD = standard deviation; LVAD = left ventricular assist device; AICD = automatic implantable cardioverter defibrillator; CRT-D = cardiac resynchronisation therapy device; CABG = coronary artery bypass grafting; PCWP = pulmonary capillary wedge pressure; BUN = blood urea nitrogen; Na = sodium; SGOT = serum glutamic oxaloacetic transaminase; SGPT = serum glutamic pyruvic transaminase.

All patients’ pre-operative data (catheterisation, echocardiography, laboratory and infection parameters) and haemodynamic status had been assessed at the hospital’s medical review board meeting to evaluate their selection for LVAD implantation. All patients had signed informed consent and the study protocol was approved by the institutional review board.

In December 2011, 55 serial two-dimensional transthoracic echocardiographic (TTE) examinations had been performed on these patients with the Vivid 3 (General Electric, Fairfield, Connecticut), and 16 serial three-dimensional TTE examinations had been done with the Philips IE33 xMATRIX (Royal Philips Electronics, Amsterdam, Netherlands). Nine transoesophageal echocardiograms (TEE) were done in the operating room under general anaesthesia during the LVAD implantation to evaluate inflow cannula and septum position, and to monitor the de-airing process while weaning from cardiopulmonary bypass. The specific protocol used for these echocardiographic studies included standard TTE parasternal, apical, subcostal and suprasternal notch views.^1^,^2^,^3^,^4^,^5^,^6^,^7^,^8^,^9^,^10^,^11^,^12^,^13^

All TTEs and TEEs were performed by certified cardiologists in different clinical settings. In hospital during the pre-implant period, they evaluated the intracardiac structure [patent foramen ovale (PFO), atrial septal defect (ASD)], chamber dimensions, left ventricular (LV) and right ventricular (RV) function, valvular structure and function, pericardial disease, volume status and abnormalities of the aorta. In hospital during the post-implant LVAD period, the aim was to visualise the septum and inflow cannula position, the intracardiac volume, outflow conduit, decompression of the left ventricle with rpm speed change and its effects on the aortic valve opening time. Doppler interrogation of the inlet cannula and outflow conduit flows were performed as described in the literature.^10^,^12^,^13^ Follow-up TTEs were done for clinical indications, including LV and RV ejection fraction (EF), RV fractional area contraction (RVFAC), unexplained change in haemodynamic status including minor dehydration, ventricular tachycardia attacks, suspicion of device malfunction, excess current alarms, and optimisation of LVAD speed.

Echo-specific parameters and differences in image quality or artifact generation were not evaluated in our study. During all echocardiographic studies, the transplant surgeons were at the bedside or in the out-patient clinic, in communication with the heart failure cardiologist setting up the pump parameters, optimising the LVAD speed, and observing the aortic valve opening times and septum inflow cannula position to prevent the suction cascade.

Images from 55 TTEs were retrospectively analysed by the heart failure cardiologist who had mainly performed all these echo studies. The inlet cannula/outflow conduit velocities were measured in m/s by spectral Doppler. The pulsality index of the pump, LV end-diastolic dimension (LVEDD), LV end-systolic dimension (LVESD), interventricular septum (IVS), posterior wall thickness, RVFAC, and tricuspid annular plane systolic excursion (TAPSE) were measured, and the functioning of the valves was routinely evaluated pre-implant and at the second week, and first, third, sixth, ninth and 12th months post-implant.

## Statistical analysis

All echocardiographic data were collected in parallel with the patients’ laboratory parameters (biochemical, blood count, coagulation profile) in an electronic database, and descriptive statistics were calculated using Microsoft Excel (Microsoft Corp, Redmond, Washington). Continuous variables were expressed as the mean value ± standard deviation.

## Results

As our Heart Assist 5 LVAD patient data were limited, we did not compare the patients’ outcome parameters pre- versus postimplant, knowing that with this small group, all analyses would be statistically non-significant. We therefore analysed these patients’ data as descriptive statistics.

**Fig. 4:**
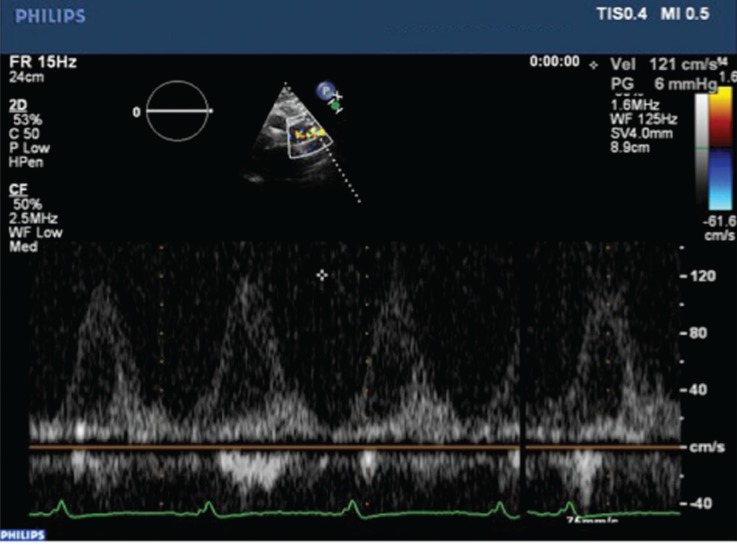
Outflow cannula velocity measurement with Doppler.

**Fig. 5:**
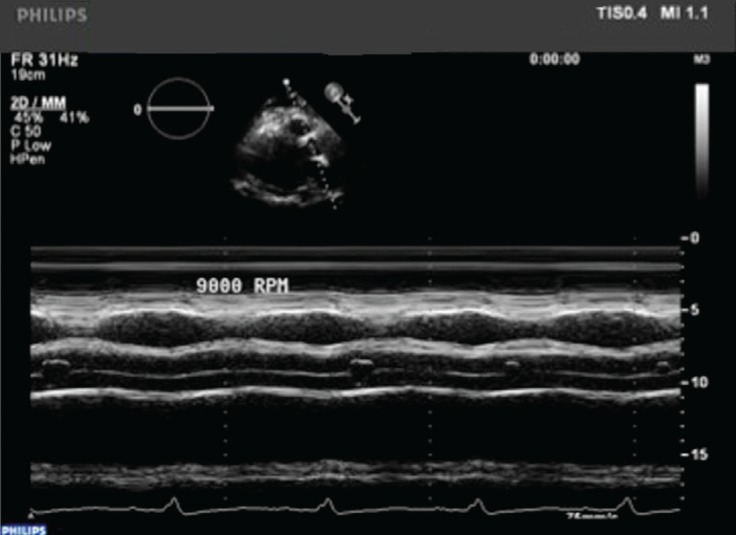
Evaluation of aortic valve opening times with M mode TTE.

**Fig. 6:**
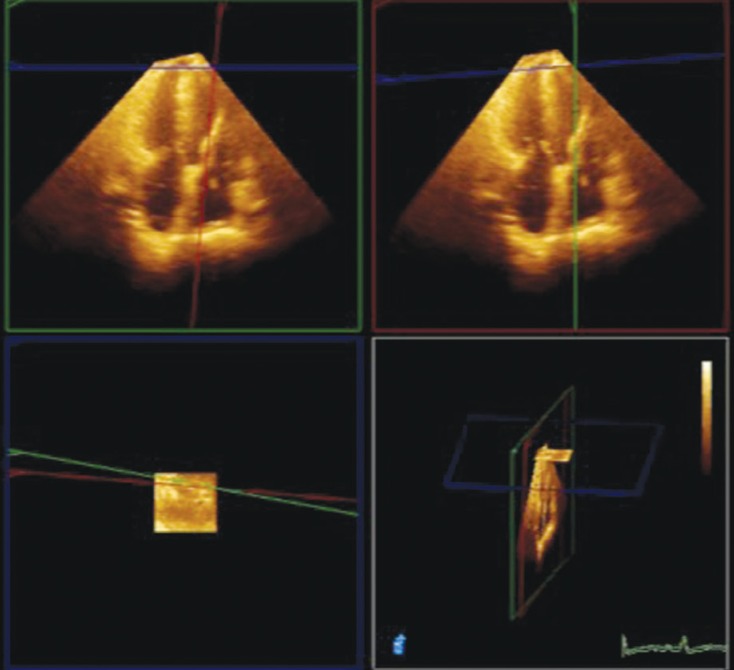
Left ventricular inflow cannula visualisation with 3D TTE.

The titanium impeller of Heart Assist 5 LVAD is small, lightweight and located pericardially. The titanium housing enables direct visualisation of the impeller. The outflow and inflow cannulas were visualised in all studies. The inflow cannula and septum (left, right, neutral) position, outflow graft anastomosis to the aorta, aortic valve cusp status, and aortic valve opening times with speed changes were evaluated and visualised in all 55 echocardiographic studies (Fig. 4)(Fig. 5)(Fig. 6).

Pre-implant echocardiographic data of the six patients: mean EF was 23 ± 5 (18–28)%, mean LVEDD was 6.9 ± 0.6 (6.3–7.7) cm, mean LVESD was 5.8 ± 0.5 (5.1–6.4) cm, mean IVS was 0.9 ± 0.1 (0.9–1.1) cm, mean RVFAC was 43 ± 9 (35–55)%, and mean TAPSE was 17 ± 4 (13–23) mm.

The 12-month post-implant echocardiographic data of the six patients were: mean EF was 19 ± 6 (10–25)%, mean LVEDD was 6.4 ± 0.4 (6.1–7.0) cm, mean LVESD was 5.6 ± 0.3 (5.2–6.0) cm, mean RVFAC was 35 ± 11(21–43), mean TAPSE was 13 ± 2 (11–16) mm, mean rpm was 9 800 ± 600 (9 500–10 400) rpm, mean pulsality index (PI) was 2.79 ± 1.7 (1.9–4.9) m/s, outflow cannula velocity was 0.9 ± 0.2 (0.8–1.2) m/s, and aortic valve opening was observed every one-to-one cycle or one-to-three cycle.

Since eight patients had ischaemic cardiomyopathy and one had had coronary artery bypass grafting (CABG) 10 years earlier, remission of their end-stage heart failure was not expected. All our patients had had automated internal cardioverter defibrillator (AICD) and cardiac resynchronisation therapy (CRT-D) implantation prior to the LVAD implantation. One patient had a ventricular fibrillation (VF) event in the ward and had cardiopulmonary resuscitation. He had his AICDCRT- D implanted prior to the LVAD surgery. Two patients had experienced ventricular tachycardia (VT) at home due to dehydration during the summer, and their AICD had been interrupted during the attacks.

There were a few hospitalisations because of minor complications. One patient complained of a coffee-grinding noise coming from his pump three months post-implant, and the remote monitoring system also documented frequent excess current alarms and increased power at different hours of the day. The patient was hospitalised for possible thrombus formation or device malfunction. The data were analysed at Micromed Cardiovascular Inc, Houston, Texas. The coagulation profile was in the normal range for a LVAD patient, his INR was 3.2, and his lactate dehydrogenase level was 606 U/l, which was gradually elevated up to 120 days after LVAD implantation.

Serial echocardiographic studies were done weekly to rule out possible thrombus formation in the left ventricle, the orifice of the inflow cannula, outflow conduit and aortic valve cusps. The patient received heparin infusion with close monitoring of his coagulation profile. There was no absolute finding in the echo studies to confirm thrombus formation or determine the reason for the coffee-grinding noise, since we were not able to visualise the titanium impeller. We guessed that there may have been a blood clot there, which caused the increased power. The noise stopped after the fifth day of heparin infusion.

A female patient had uncontrolled diabetes, she had gradually gained 7 kg by the sixth month post-operatively, and suffered from recurrent urinary tract infections. She was admitted with fever, tachycardia, post-diabetic ketoacidosis and documented urinary tract infection. Her echocardiographic study was done in a sub-optimal position due to her tachycardia. She received anti-arrhythmic and antibiotic regimens, and a few days later she was in sinus rhythm, her heart rate was 89–95 beats per minute, and her fever was gone. Her echo studies were repeated and LVAD optimisation was done. She was discharged from hospital with 9 500 rpm, her CO was 4.7 l/min and the pulsality index was 2.31 m/s.

Four patients were followed up for more than two years, and two for more than a year. Three patients died due to multi-organ failure. One international patient had heart transplantation in the Republic of China. The longest supported patient was for more than three years, with a good quality of life.

## Discussion

All data for each patient had been collected in each portable console, Heart Attendant (Micromed Cardiovascular Inc, Houston, TX), so with the universal serial bus (usb) connection, all the data could be downloaded and could be easily analysed. Online connection of the Heart Attendant to the internet assists in patient care and provides 24 hours a day, seven days a week monitoring of the LVAD patient. This technology decreases hospital admissions for each alarm detection, as the clinicians can track the patient with the remote monitoring system and help him/her by phone.

This technology differentiates Heart Assist 5 LVAD from other continuous-flow pumps. The implantable flow probe also helps to assess even minor dehydration problems, which can be solved without admission to hospital. We experienced low-flow alarms during the summer months and suggested our patients take in extra fluid during summer.^15^ Fine-tuning of the Heart Assist 5 LVAD with the implantable flow probe and portable console was convenient, and the LVAD speed change echo studies enabled easy optimisation of the device during in-hospital echocardiographic studies.

There are reports of large single-centre echocardiographicstudies of other continuous-flow and centrifugal pumps in the literature.^8^,^9^,^10^,^11^,^12^,^13^ These pumps have been approved by the United States Food and Drug Administration (FDA) and Conformitė Europėenne (CE), and have been on the market for a long time. Many centres have implanted these pumps in their end-stage heart failure patients all over the world.

Long-term outcomes of these pumps have been published recently in the literature.^1^,^2^,^3^,^4^,^5^,^6^,^7^,^8^,^9^,^10^,^11^,^12^,^13^ Frazier *et al* demonstrated the modified Micromed axial flow pump has an in vitro responsivity approaching that of the natural heart.^16^ Bluestein *et al* reported that the thrombogenicity of Heart Assist 5 was 2.5-fold lower than the HeartMate II LVAD.^17^

Although our experience with Heart Assist 5 LVAD was not from a large series, compared with other continuous-flow and centrifugal pumps that we have implanted in our clinic, we tried to standardise pre-operative LVAD evaluations and post-implant follow up of LVAD patients in collaboration with the heart failure cardiologists. We believe that as a heart failure/transplant team, a systematic approach for the evaluation of these patients suffering from advanced heart failure is important. Close follow up of these patients will affect the success of LVAD surgery and long-term prognosis of the patients, which will lessen the side effects and enable rapid and accurate identification of mechanical and systemic malfunctions.

Our group was the first to implant this version of Heart Assist 5 LVAD in Turkey. An earlier version of this pump was implanted in Turkey in April 2001.^14^ There were no data in the literature on the Heart Assist 5 LVAD using echocardiographic parameters and LVAD optimisation protocols during long-term follow up of the patients. In our clinical studies we were able to visualise the inflow cannula and outflow graft anastomoses to the aorta in most of the echocardiographic studies. The remote monitoring system and the implantable flow probe measurements were reliable, and tracking and monitoring of these patients outside of hospital was easy.

Our study has limitations since there were so few patients with Heart Assist 5 LVAD support in our group. However, if other centres start implanting Heart Assist 5 LVAD in their end-stage heart failure patients, more data on these pumps and their longterm outcome will be available for clinicians in the future. This pump has had CE approval in Europe since May 2009, and the National Health Government solved the reimbursement issues of these continuous-flow pumps in 2012.

## Conclusion

Our study was not designed for the appropriate combination of flow, power, pulsatily index and left ventricular unloading. It is impossible to establish or document cause–effect relationships while optimising LVAD parameters with so few patients. This study was an observational analysis of a new-generation pump. We believe that a concept should be studied in larger prospective series with different pumps using serial echocardiographic studies and comparing long-term outcomes of each pump.
